# Mental rotation, perspective taking, and performance profiling

**DOI:** 10.1007/s10339-025-01269-6

**Published:** 2025-03-28

**Authors:** James Negen

**Affiliations:** School of Psychology, Liverpool John Moores University, Liverpool, UK

**Keywords:** Spatial cognition, Typology, Mental rotation, Perspective taking, Intrinsic spatial cognition, Extrinsic spatial cognition

## Abstract

In spatial cognition, we conventionally draw a typological distinction between mental rotation (intrinsic, object movement) versus perspective taking (extrinsic, self movement). This paper re-examines a previous finding which could indicate that fundamentally different cognitive processes are reflected in these tasks. Specifically, performance as a function of rotation magnitude is a linear profile for mental rotation but a notched profile for perspective taking. Experiment 1 conceptually replicates this, finding a task by rotation magnitude interaction with more participants, more trials, and updated statistical controls. Experiment 2 extends the previous analysis to verify that the two performance profiles are genuinely different shapes rather than different effect sizes. Together these help confirm that mental rotation and perspective taking reflect fundamentally different cognitive processes, thus justifying their central focus in the typology of spatial cognition.

## Introduction

Typology is a fundamental part of any complete, useful understanding of a subject area. It creates a natural flow and organization of teaching and learning. This extends into organizing research too—and if it is done carefully, it can extend further into organizing/informing treatments and interventions as well. This is the happy state we are beginning to see in spatial cognition, which has seen several related proposals for typology (Newcombe 2018; Newcombe & Shipley 2015; Uttal et al. 2013). These theories organize spatial cognition into kinds so that each can be studied and understood. Further, these theories have directly informed the creation of different strategies for spatial training with remarkable effects on math and science learning from interventions that last mere minutes (Gilligan et al. 2018, 2020). In other words, a typology of spatial cognition is important because it is useful for understanding spatial cognition and it also has practical implications.

**Fig. 1:**
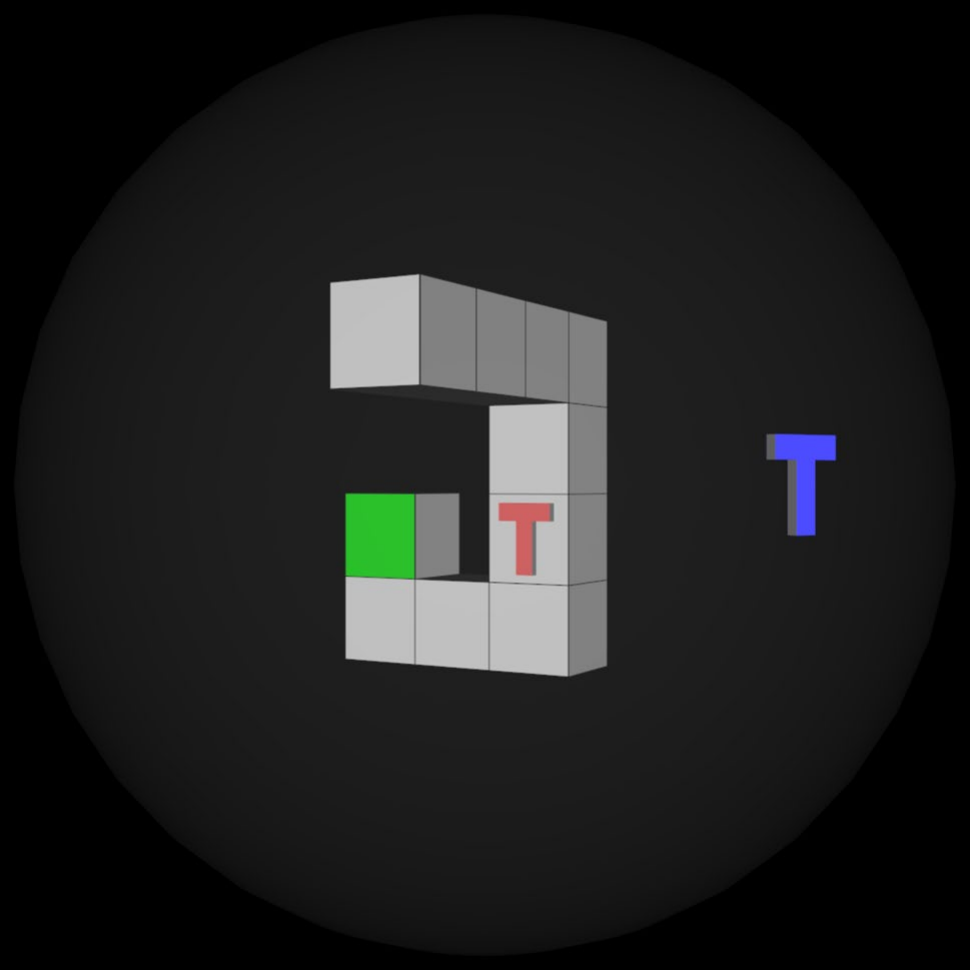
Example stimulus for the task. For the extrinsic/perspective-taking/self-movement task, imagine your perspective rotating so that you are looking through the blue T. For the intrinsic/mental rotation/object-movement task, imagine the object rotating until the red T faces the blue T. For both, try to work out if you would still be able to see the green square. Note that any algorithm that can rotate the scene would be able to solve both tasks

A previous paper (Wraga et al. 2005) reported a specific finding that could be very important evidence for the typology of spatial cognition. They were studying imagined rotations of self versus object, more commonly referred to as a perspective taking task versus a mental rotation task, which the theories above map onto extrinsic versus intrinsic spatial cognition (Newcombe 2018; Newcombe & Shipley 2015; Uttal et al. 2013). See Fig. 1 for an example with the stimuli used here. When charting performance (i.e. reaction time, accuracy) as a function of the rotation magnitude, they found two very different patterns. Performance fell as rotation magnitude increased for the object moving. However, performance actually improved for the middle rotation versus the smallest rotation in the self moving condition, then fell again for the highest rotation. This can only be explained by positing a completely different cognitive process—one process that works in increments so that performance degrades as rotation magnitude increases versus another process that evaluates relative magnitudes in a way where a bigger rotation magnitude can actually be easier. This finding stands out for being very direct (it avoids all the pitfalls of interpreting correlations to other tasks and variables), very well controlled (stimuli were nearly identical; within-subjects design), and very helpful towards limiting the possible theories of the underlying cognitive process (cognitive models must reproduce similar patterns). Further, if we can verify it in detail, then it will suggest the performance profiles are a completely different shape (not just different effect sizes), indicating qualitatively different cognitive processes. This could be viewed a cornerstone finding for the typology reviewed above; at least, it deserves careful verification.

The central goal of this project is to see if we can conceptually replicate this finding, verify the different performance profile shapes in detail, and also update the methods and analysis where opportunities arise. If so, it will solidify the evidence that these seemingly minor task differences tap into fundamentally different cognitive processes being carried out for the same spatial layouts, justifying their place as a central part of the typology of spatial cognition.

### Context and background

Of course, the key finding discussed above is part of a larger research context that has found various reasons to dissociate the typical perspective taking versus mental rotation tasks. There can be differences in their overall difficulty (Huttenlocher & Presson 1973, 1979; Wraga et al. 2000, 2004). They tend to activate different brain areas (Ratcliff 1979; Wraga et al. 2005; Zacks et al. 1999, 2003). They tend to load as different factors (Hegarty & Waller 2004; Kozhevnikov & Hegarty 2001; Mix et al. 2018). There can be different developmental trajectories (Hodgkiss et al. 2021). They can relate in different ways to spatial anxiety or self-reported spatial skill (Hegarty et al. 2006). They can predict different education outcomes (Gilligan et al. 2018, 2020). They can show different gender effects (Hegarty et al. 2006; Voyer et al. 1995). This all means that a given researcher or practitioner might have reason to separate the two even if they do not particularly have an interest in the underlying cognitive processes.

There is also research that draws conclusions towards a difference in the underlying cognitive process. There are a variety of findings that are broadly in line with the key one described above—they report some form of task by rotation magnitude interaction (Presson 1982; Wang & Simons 1999; Wraga et al. 2000, 2004) or at least imply it through a pattern of significance and non-significance (Huttenlocher & Presson 1973, 1979; Simons & Wang 1998). These findings are less pointed at a difference in profile shape, rather than having the same shape with different effect sizes, but they still might be considered evidence for a cognitive difference. In addition, researchers may see results in the paragraph above as pointing towards a difference in cognitive processes, depending on their perspective.

The extrinsic versus intrinsic divide is also cross-cut by a distinction between static and dynamic spatial cognition to complete a central four-part typology. Static deals with creating representations, such a spatial scaling task (extrinsic static) or an embedded figures task (intrinsic static), whereas dynamic deals with manipulating representations, like the perspective taking task here (extrinsic dynamic) and the mental rotation task here (intrinsic dynamic). There is also a kind of offshoot type, the use of space as symbol, which includes things like the use of spatial gestures and metaphors to indicate important relations that are not literally spatial. Any reader who is interested in this wider typology should see existing detailed reviews of the area (Newcombe 2018; Newcombe & Shipley 2015; Uttal et al. 2013).

### The present study

The finding being re-examined here needs such re-examination to be sure that it replicates and to meet the best possible practices for the method and analysis. The study in question (Wraga et al. 2005) was not primarily designed to examine behavioural performance profiles. To accommodate their fMRI work, they used a modest sample size and modest number of trials. Experiment 1 aims primarily to conceptually replicate their findings with a larger sample and more trials. The updated, pre-registered analysis also deals explicitly with speed-accuracy trade-offs and multiple comparisons. In addition, the previous paper’s analysis stopped just short of confirming that the performance profiles were genuinely different shapes. They showed a task by rotation magnitude interaction, but were not able to verify that this did not just reflect a stronger rotation magnitude effect in one task than the other (i.e. the mental rotation version may just attenuate the rotation magnitude effect somehow, rather than reflecting a fundamentally different shape and thus cognitive process). Experiment 2 again replicates the previous finding and extends this to verifying a difference in performance profile shape.

## Experiment 1

Experiment 1 addresses the existence of the rotation magnitude by task interaction, replicating previous research with some updates to the methods and analysis (more trials, more participants, pre-registered analysis that directly addresses speed-accuracy trade-offs and multiple comparisons). The main hypothesis was a rotation magnitude by task interaction in terms of performance. The secondary hypothesis was a more specific version of the main hypothesis—specifically, that the mental rotation task will show a linear effect while the perspective taking task will show a flat profile except for a notch of high performance at 90°. This would be reflected in each task having significant simple effects of rotation magnitude, the rotation magnitude simple effect for the mental rotation task being linear, and the rotation magnitude simple effect for the perspective taking task attenuating to non-significance when the 90° turns are removed.

### Method

Experiment 1 was pre-registered at https://osf.io/6dmc7. The full methods and the resulting data are posted on the associated project at https://osf.io/5k6gu/.

#### Participants

There were ultimately 20 participants (5 male, 13 female, 2 preferred not to say; min 27 years, max 57, mean 38, standard deviation 9). They were recruited through Prolific by screening for English fluency and normal or corrected-to-normal vision. An additional 13 participants failed to meet the pre-registered 75% correct response rate for inclusion. All participants (even those whose data were excluded) were paid £4. The study was approved by Liverpool John Moores University’s UREC (Reference: 22/PSY/025).

The plan was to recruit up to 40 participants with an optional stopping rule. This final goal number was based on the small telescopes approach (Simonsohn 2015) which advocates replicating an effect with 2.5 × the number of participants. This would mean 12 × 2.5 = 30 participants. This was increased to 40 to give some allowance for any unforeseen difficulty in measuring the effects of interest. The recruiting method was selected to balance the ability to have many participants for high power against the potential outcome where only a few are needed. To balance this, the O'Brien Fleming optional stopping method for 4 looks (O’Brien & Fleming 1979) was employed. This involves collecting data from 10 participants and testing it against α = 0.00005. If the key outcome is significant (magnitude by task interaction), testing stops. If not, another 10 are collected and tested against α = 0.0039. As before, if significant, testing stops; if not, this proceeds with α = 0.0184 and finally α = 0.0412. One can think of this as ‘spending’ alpha in phases so that it may be possible to stop early if a large sample is not required. This procedure preserves the type I error rate and has a negligible impact on statistical power while allowing for fewer participants.

#### Apparatus

Participants completed the testing on their computers or touchscreen tablets. During the trial, several things were on screen: the stimulus (described below), a green 'YES' button, a red 'NO' button, and brief instructions: "Imagine YOU MOVED until you were square with the blue T. Could you see the green square?" or "Imagine the OBJECT TURNED until the Ts faced each other. Could you see the green square?".

#### Stimuli

Each stimulus had a few essential components. The central object was a set of 10 cubes arranged into a contiguous object. One cube face was green. One cube face had a red T on it. The entire cube object is rotated 30 degrees left from the camera, making the front and right sides visible. The green square and T were always on one of these visible cube faces. There was also a low-opacity sphere around the object. On the surface of the sphere was a blue T that faced towards the center of the sphere.

**Fig. 2:**
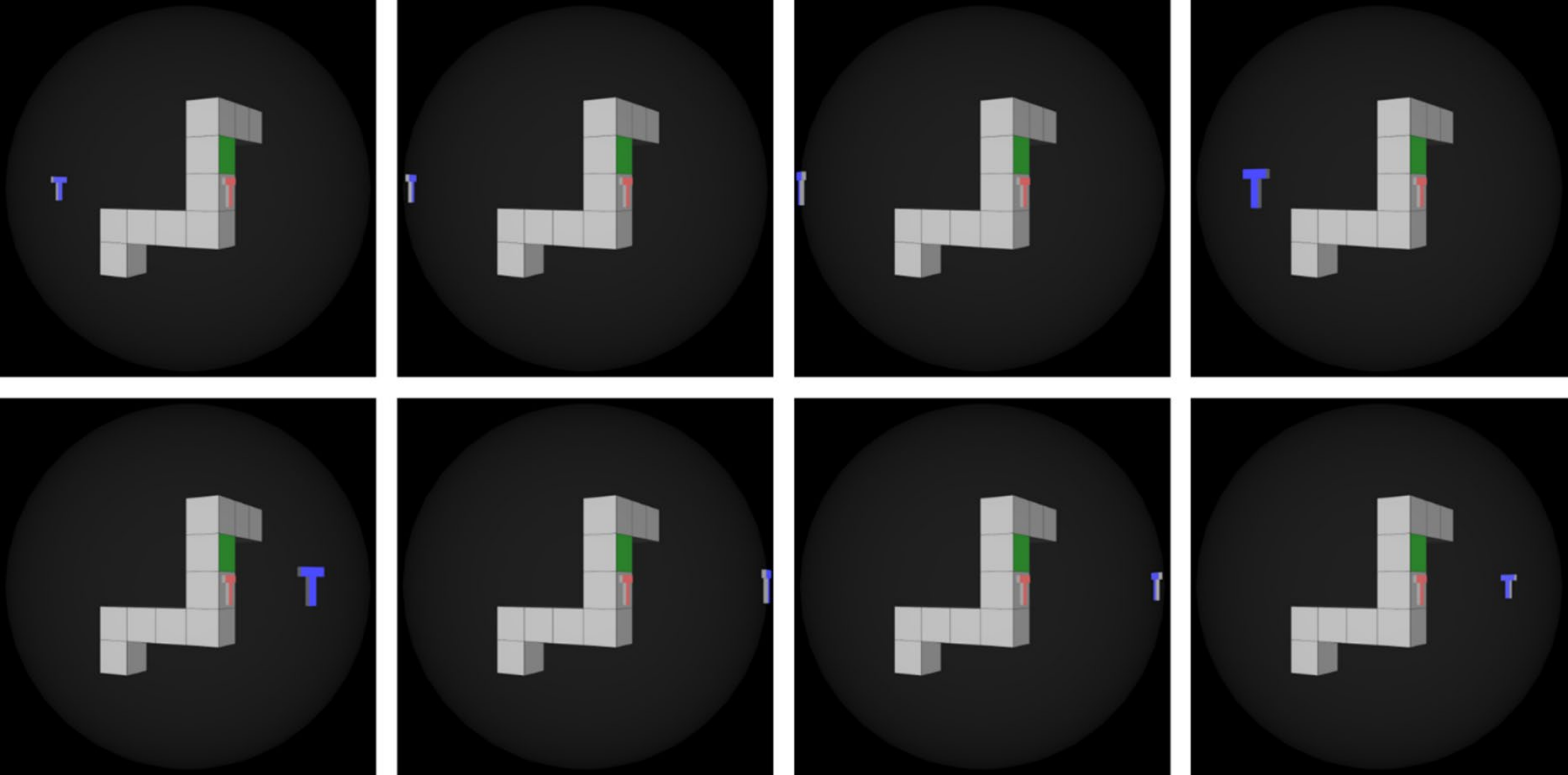
All 8 renderings of the blue T for Object 1

There are a total of 20 central objects. Each was rendered 8 times (Fig. 2): the blue T was rendered at − 120, − 90, − 60, − 30, 30, 60, 90, and 120 degrees from the camera position. It did not ever occlude the central object. The side for the red T (left versus right) and green square (left versus right) were counterbalanced so that each of the four possible combinations was present once within each testing phase. These were produced at 1080 × 1080 resolution.

#### Procedure

**Fig. 3:**
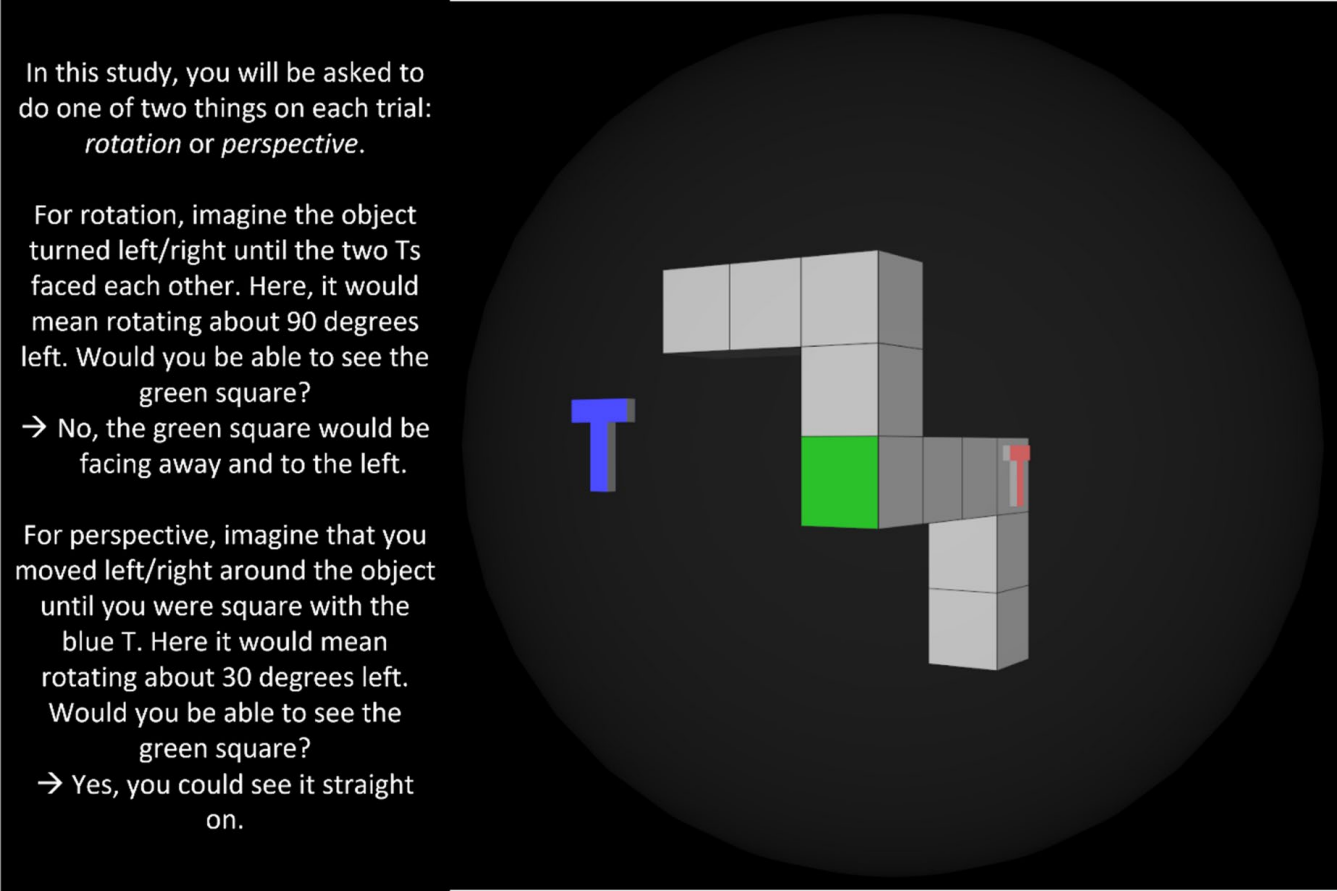
Instructions given to participants with an example stimulus

To begin, an instruction slide was shown (Fig. 3). This gave an example stimulus and walked through how to do each of the two tasks to get the correct answer.

The main procedure formed into four superblocks. Each superblock had two blocks: one perspective taking block then one mental rotation block. Each block had two phases: practice and testing. Practice phases constituted 8 trials with a single object. Practice phase data were not analyzed. Testing phases constituted 32 trials with 4 objects (8 each). Order within each phase was random.

Each trial was done simply by showing the participant the stimulus and making the responses available. If the response was correct, the next trial began immediately. If not, it paused for three seconds with the stimulus visible.

Between blocks, an image indicating a task change appeared for five seconds. This was a static image of an exclamation mark in a triangle with the text “Task Changing” beneath it.

#### Planned analysis

The central goal was to analyze for a magnitude by task interaction. The outcome was meant to be a sense of performance on the task. The pre-registration had a specific plan to deal with speed-accuracy trade-offs, beginning with a check to see if such a trade-off was occurring and possibly driving the interaction. If needed, the pre-registered plan was to use the inverse efficiency score (Townsend & Ashby 1983). In short, this check did not find evidence that this was a concern for these results; correcting for possible trade-offs would only exaggerate the interaction effects reported below. As such, no speed-accuracy correction was applied for this experiment. Because each outcome (reaction time, accuracy) could trigger the stop, each was given half of the nominal alpha value. The main analyses consisted of two 4 (rotation magnitude: 30°, 60°, 90°, 120°) by 2 (task: perspective, rotation) repeated-measures ANOVAs, with one ANOVA for accuracy and one ANOVA for reaction time.

#### Outlier exclusion and processing

There were three layers of exclusion and processing. First, any participant with a correct response rate below 75% was entirely excluded. This rate was calculated with the 256 analyzable trials (i.e. the testing phases). Second, reaction time was calculated in 8 cells per participant (30°, 60°, 90°, or 120° magnitude; mental rotation versus perspective taking) as the median of all relevant reaction times when the response was correct. This should dampen the effect of any odd trial that took a very long time due to things like computer malfunction. In the same 8 cells, percent correct was also recorded. Note that this means some mental rotation data is not used as some mental rotation magnitudes did not have any data from matching perspective taking magnitudes (specifically 0°, 150°, and 180°). Third, there was outlier exclusion done separately on the central outcomes (RT, accuracy). Outcomes were centered within each participant to have a mean of zero (i.e. main effect of participant removed) since the analyses are within-subjects. If a participant had any aggregate data in their 8 cells that was more than 3 SDs above or below the group mean, that participant's data was excluded for that outcome. This led to 1 exclusion for RT and zero for accuracy.

### Results

#### Main analysis

**Fig. 4:**
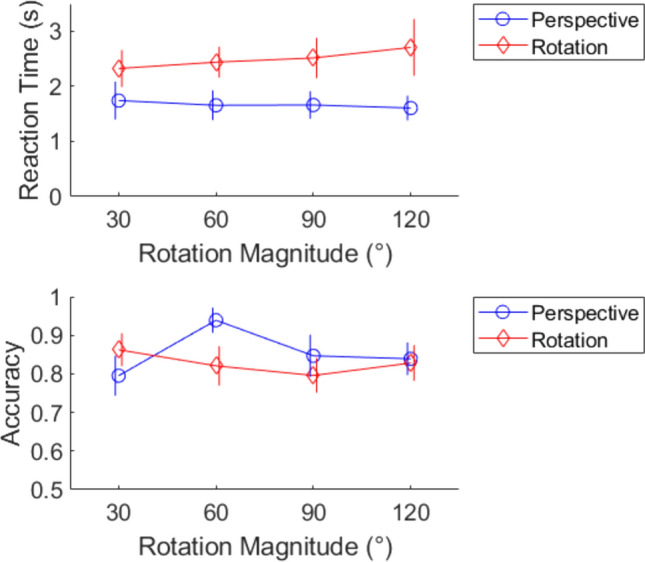
Performance in terms of rotation magnitude, task, and performance outcome. The accuracy measure in particular triggered the stopping rule with a magnitude by task interaction, *p* < .001

The main hypothesis was supported. There was a significant rotation magnitude by task interaction in terms of accuracy, F(3, 57) = 11.95, *p* < 0.001 against an adjusted α = 0.00195, η^2^ = 0.101 (Fig. 4). No speed-accuracy trade-off correction was applied as this would only exaggerate the effect. This confirms that rotation magnitude has different effects on the mental rotation versus perspective taking task, conceptually replicating previous results.

#### Secondary hypotheses

The secondary hypothesis was partially supported. The mental rotation task predictions were correct. There was a significant main effect of rotation magnitude for the reaction time outcome for the mental rotation task, F(3, 54) = 3.09, *p* = 0.035, η^2^ = 0.031, though not also for accuracy, F(3, 57) = 2.60, *p* = 0.061, η^2^ = 0.057. This was not significantly different from a linear effect, F(3,54) = 0.106, *p* = 0.956, η^2^ = 0.001. This is consistent so far.

The perspective taking task predictions were mixed. With reaction time as the outcome, rotation magnitude did not have a significant main effect, F(3, 54) = 1.03, *p* = 0.386, η^2^ = 0.007, but did have a significant effect for the accuracy outcome, F(3, 57) = 10.8, *p* < 0.001, η^2^ = 0.226. This is consistent so far. However, this was still significant when removing the 90° data, F(2, 38) = 18.8, *p* < 0.001, η^2^ = 0.309. This is not consistent with the secondary hypothesis. Inspection of Fig. 4 shows why this is the case: the main effect is mainly driven by the difference between 60° versus the others. Unexpectedly, the main effect of rotation magnitude is non-significant when instead removing the 60° data, F(2, 38) = 1.96, *p* = 0.155, η^2^ = 0.045. This is still a ‘notch’ pattern but not the expected one.

This is generally consistent with an interpretation of the mental rotation task as a linear process and the perspective taking task as a process with preferred directions—but not the specific idea that those preferred direction are always cardinal to the observer.

### Discussion

The results from Experiment 1 verify that there is a rotation magnitude by task interaction, even with the updated methods and analysis. This included more trials, more participants, an explicit check against speed-accuracy trade-offs, and explicit corrections for multiple comparisons. This at least confirms that the same manipulation leads to different effects on performance for the two tasks.

With that said, the argument for a performance profile shape difference requires further work. Analysis available is consistent with a difference in shape (linear versus notched). However, the discovered notch shape is not consistent with the pre-registration. More importantly, parts of the argument above for a shape difference rely on non-significant findings being interpreted as non-differences (i.e. since the mental rotation reaction times do not deviate significantly from linear decreasing, they are linear decreasing). This is not up to the highest standards of evidence. Using the results from Experiment 1 as a guide we can pre-register a new experiment that can create a full positive case for a qualitative difference in performance profile shapes if its predictions are confirmed.

## Experiment 2

Experiment 2 is designed to build on Experiment 1 to see if it can be replicated and also to see if the interaction can be properly understood as a performance profile shape difference. To best facilitate this, several changes were made.

First, to keep this manageable, the outcome was switched entirely to balanced integration score (BIS). Previous work shows that this surprisingly tractable measure, the z-score of the percent correct minus the z-score of the reaction time, accounts well for speed-accuracy tradeoffs and creates a singular performance measure (Liesefeld & Janczyk 2019). The result is unitless but easy to understand: higher is better. By using a single outcome that already accounts for both speed and accuracy, there is no need for further speed-accuracy trade-off controls. All predictions in Experiment 2 are in terms of BIS.

Second and most importantly, the exact difference in the two profiles was pre-registered as an extended prediction. For experiment 2, the prediction will be considered confirmed if and only if all seven of the following are found: (1) a rotation magnitude by task interaction; (2) a simple effect of rotation magnitude for the mental rotation task; (3) a simple effect of rotation magnitude for the perspective taking task; (4) 60° > 30° for the perspective taking task; (5) 60° > 90° for the perspective taking task; (6) 30° > 60° for the mental rotation task; (7) 60° > 90° for the mental rotation task. If all of these are found, this will systematically rule out (a) that the two profiles are the same via 1; (b) that either is flat via 2 and 3; and (c) that their shapes are the same by showing that one is a notch and the other is decreasing via 4 through 7. If found, this will positively verify the supposition that mental rotation and perspective taking tasks, when closely matched, still result in qualitatively different performance profiles. Further comparisons to 120° were left out since they are not strictly needed to show a shape difference and each additional requirement lowers the statistical power.

Third, in keeping with the advantages of the BIS, the inclusion criteria were relaxed. Since the BIS is known to account for speed accuracy trade-off issues over a wider range of accuracy, we can relax the inclusion so as to merely screen away participants who do not understand the task. This makes the result more reflective of a wider population. The new exclusion criteria are (a) overall accuracy below 60% or (b) any BIS score more than 2.5 standard deviations from the overall mean for the same magnitude and task.

Fourth, to be as careful as possible about the matching between tasks, the set of trials entered into the analysis was further restricted. Specifically, a trial was only used in the analysis if the exact same object also had a trial of the other task with the exact same final view in the solution. In other words, if one trial requires object 10 to be rotated 30° to the right in the mental rotation task, that trial will only be included if there is another trial where object 10 needs to be rotated 30° to the right in the perspective taking task. This is moderately stricter than Experiment 1 which treated either 30° rotation, left or right, within the same task as interchangeable for matching purposes. There are now 176 included trials per participant (48 at 30°, 60°, and 90°, plus another 32 at 120°).

For comparison later, here are the results of Experiment 1 re-done with these changes to the analysis: 24 participants were included. There was a significant rotation magnitude by task interaction, F(2, 69) = 9.21, *p* < 0.001. Each simple effect of rotation magnitude was significant, F(3, 69) = 7.86, *p* < 0.001 for perspective taking and F(3, 69) = 4.75, *p* = 0.005 for mental rotation. The notch pattern was significant in the perspective taking data, *p* < 0.001 and *p* = 0.036 when comparing 60° against 30° and 90°. The decreasing pattern was not confirmed in the mental rotation data, *p* = 0.053 and *p* = 0.089 when comparing 30° to 60° and 60° to 90°. If this were the final result of Experiment 2, this would be taken as failing to confirm that there is a qualitative performance profile shape difference present. Specifically it leaves open the possibility that the interaction reflects a attenuated version of the perspective taking shape for the mental rotation task (i.e. the same pattern of upward and downward movements, just with lower slope).

The fifth and final change is that two object stimuli were re-worked to eliminate any ambiguity. An anonymous participant sent a message to point out that since two objects have a green square facing the center of the sphere, a very small portion of the green square could still be visible after one of the rotations that was marked with no as the correct answer. While the effect of this is likely negligible, there is no reason not to correct this for a second experiment.

### Method

Experiment 2 was pre-registered at https://osf.io/sk9bu.

#### Participants

The plan was to gather up to 50 participants with the O’Brien Fleming Boundary for 5 looks. Informally, as Experiment 1 nearly found all seven effects with only N = 20, up to N = 50 should be more than sufficient. Formally, with N = 50, power is 80% for d = 0.35 which is smaller than the smallest measured effect in Experiment 1 (d = 0.386 in re-analysis, 60° versus 90°, perspective task). As it happens, all 50 were gathered (plus 11 exclusions) and thus alpha is adjusted to 0.0417. There were 34 men, 15 women, and one who preferred not to say (20 to 65 years old, mean of 34.1, SD of 11.1).

#### Apparatus, stimuli, and procedure

These are kept consistent with Experiment 1 with one exception. Two objects were re-made so that the green square did not face the center of the sphere. New stimuli are on OSF.

#### Planned analysis

The central goal was to confirm a detailed profile shape difference as laid out in seven parts below. The key outcome is called a Balanced Integration Score (BIS). It is the z score of the percent correct minus the z score of the reaction time. Previous research suggests that this has good ability to control for speed-accuracy trade-off issues (Liesefeld & Janczyk 2019). The overall analysis consisted of a 4 (rotation amount: 30°, 60°, 90°, 120°) by 2 (task: perspective, rotation) repeated-measures ANOVA with BIS as the outcome.

#### Outlier exclusion and processing

There were four layers of exclusion and processing. First, the relevant matched trials were selected out of each participant's data. These were all trials that were from the testing phase and also had a matching trial with the same signed magnitude on the same object but the other task. A total of 176 trials per participant met these criteria. Second, any participant with a correct response rate below 60% was entirely excluded (6 participants). This rate was calculated with the 176 analyzable trials. This was done because lower accuracy might indicate that the participant did not understand the task. Third, reaction time in 8 cells per participant (30°, 60°, 90°, or 120° rotation magnitude; mental rotation versus perspective taking) was calculated as the median of all relevant reaction times when the response was correct. This should dampen the effect of any odd trial that took a very long time due to things like computer malfunction. In the same 8 cells, percent correct was also recorded. The BIS was then calculated. Fourth, there was outlier exclusion. If a participant had any data that was more than 2.5 SDs above or below the group mean for the same task and magnitude, that participant's data were excluded (5 participants). The BIS was then re-calculated.

### Results

**Fig. 5:**
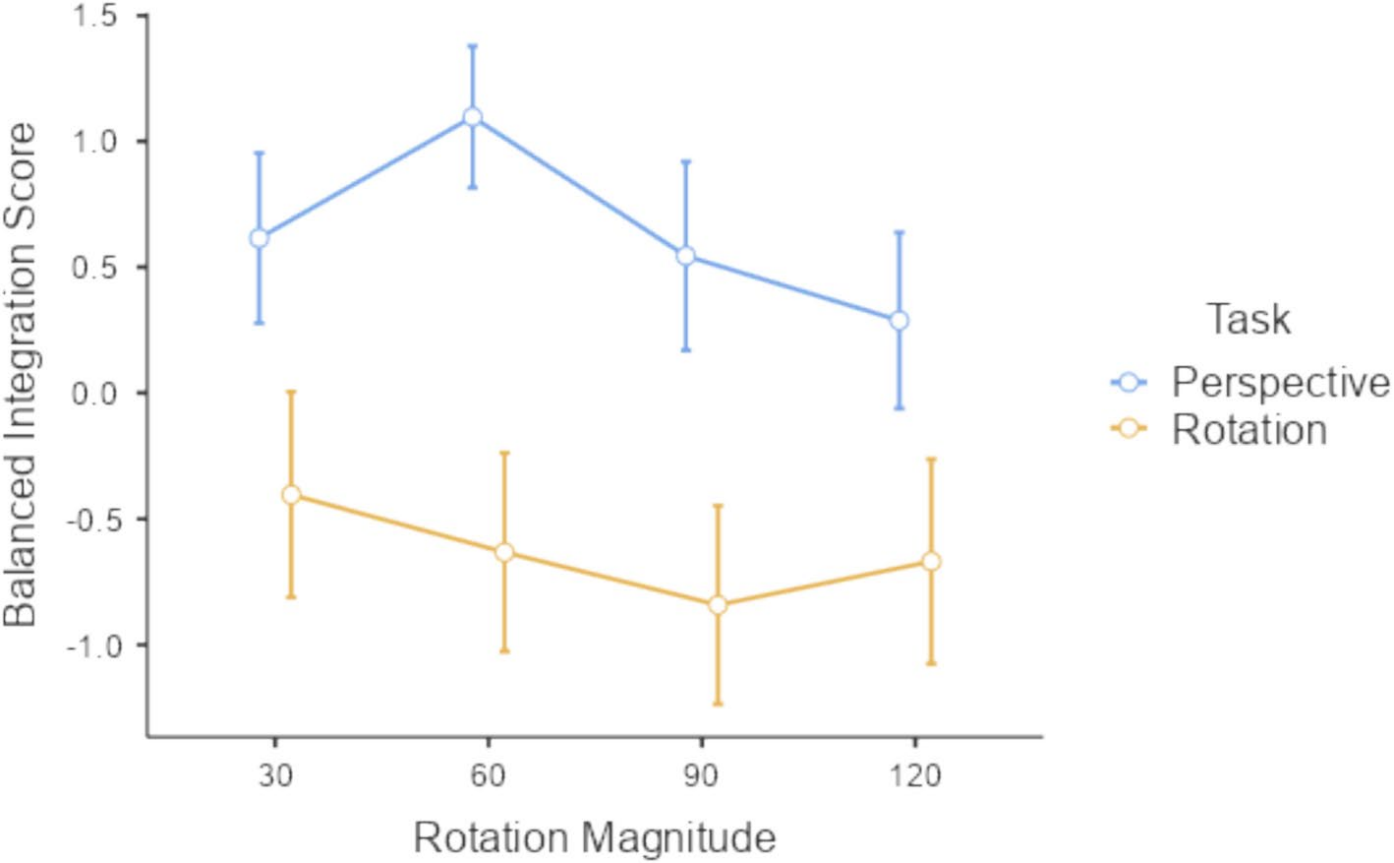
Performance as a function of rotation magnitude for the two tasks

Results confirm all seven points of the prediction with N = 50 and *p* < 0.0417 (Fig. 5). There was a significant magnitude by task interaction, F(2.81, 137.76) = 8.21, *p* < 0.001, η^2^ = 0.011 with the Greenhouse–Geisser correction. There was a significant simple effect of magnitude on the perspective taking task, F(2.63, 128.92) = 10.7, *p* < 0.001, η^2^ = 0.058, as well as the mental rotation task, F(2.94, 143.86) = 4.57, *p* = 0.005, η^2^ = 0.012. Individual contrasts in performance were also significant: 60° over 30° in perspective taking, t(49) = 3.49, *p* < 0.001, d = 0.494; 60° over 90° in perspective taking, t(49) = 3.96, *p* < 0.001, d = 0.560; 30° over 60° in mental rotation, t(49) = 1.92, *p* = 0.031, d = 0.271; 60° over 90° in mental rotation, t(49) = 1.88, *p* = 0.033, d = 0.265.

A post-hoc analysis shows that these effects all remain if the two tablet users are excluded (rows 44 and 15 in the OMV file in the Experiment 2 folder on OSF) e.g. there is still a significant magnitude by task interaction, F(2.78, 130.76) = 8.19, *p* < 0.001, η^2^ = 0.011. An additional post-hoc analysis did not find any significant interactions with gender e.g. the magnitude by task by gender interaction was not significant, F(2.81, 131.98) = 0.962, *p* = 0.408, η^2^ = 0.001.

### Discussion

By confirming all seven points of the prediction, the results suggest that the performance profile is a different shape for the two tasks. Manipulating rotation magnitude has a qualitatively different effect on performance. The mental rotation profile is decreasing, at least from 30° to 90°. The perspective taking profile is a notch (increasing then decreasing) over the same range. Oddly, the sample mean BIS actually increased from 90° to 120° for the mental rotation task, but it is somewhat difficult to say whether this is meaningful. The difference between them is not significant, t(49) = 1.36, *p* = 0.18, d = 0.193. This might be nothing more than sampling noise.

## General discussion

These two experiments developed and pre-registered a specific prediction that shows these two tasks (conventionally called mental rotation and perspective taking) react in fundamentally different ways to the same manipulation. These results are derived from the same stimulus set, the same participants, interwoven in the same session, displayed on the same devices, asking for the same response through the same mechanism, processed through the same analysis pipeline, and subject to the same statistical tests. The stimuli are even the same in terms of the central objects, the critical green faces that were the subject of both task prompts, and the final solution views. The only difference is the change in the instructions and the direction of the outer blue T (i.e. displacing it left versus right to prompt a leftwards rotation). These tasks are famously formally equivalent, yet participants did not show a similar performance profile shape. Significant results positively rule out any description of the performance profiles as (a) the same, given a significant task by rotation magnitude interaction or (b) differing only by effect size, as the perspective taking profile increased from 30° to 60° while the mental rotation decreased and then both profiles decreased from 60° to 90°. This leaves only the empirical conclusion that the performance profiles are different shapes, a decreasing function and a notch. This in turn solidifies the theoretical conclusion that there are two fundamentally different cognitive processes being tapped in these two tasks: one where performance degrades as rotation magnitude increases and one that can actually have a performance improvement for larger rotation magnitudes.

With that said, the experiments here re-used a general task design that showed a specific pattern of interest (Wraga et al. 2005), and while it does reflect similar task design in the field, it is not obvious that “intrinsic”, “mental rotation”, “extrinsic”, or “perspective taking” are the best descriptions for the exact difference reflected in the present results. These terms are re-used here because they reflect existing typology and convention. However, one thing that stands out as an alternative is the way that the mental rotation task deals with spatial relations between three items (green square and both Ts) while the perspective taking task uses only two items (green square and blue T). This is not totally unrelated to the intrinsic and extrinsic divide: when we consider the spatial relation between objects in everyday situations, we can often approximate entire objects/vistas with multiple internal parts as though they were one point/place. In terms of practical task design, any mental rotation task will always need at least three points involved—two that are on the same object and a third to fix the objective of the transformation—so it can never be reduced as far as the perspective taking task here. The result here does reinforce the conclusion that different cognitive processes are present but it also leaves open a few avenues for the best way to understand and describe the specific conditions that produce that difference.

The existing literature provides a potential explanation for the underlying processes for the two performance profiles that are reported here. The mental rotation task is classically explained as an incremental mental process (Newcombe 2018; Shepard & Metzler 1971). The idea is that people rotate a mental image of the object by small amounts. This creates a (linear) decreasing performance profile as each additional rotation takes more time and presents more opportunities for error. In contrast, the notch profile has a section where performance improves as the rotation magnitude increases, which means the underlying process must not be incremental. Instead, the overall space is understood along perpendicular axes that facilitate comparisons when aligned with the stimuli (Mou & McNamara 2002; Shelton & McNamara 2001). In other words, it is a process more akin to choosing a metaphorical “top” or “north”, relating it more to navigation (Newcombe 2018).

Stepping back slightly, it is possible that broader theories of the function of perception and cognition could account for this typological divide. From an evolutionary perspective, it is possible that the mental rotation task reflects evolving tool use and that the perspective taking task reflects navigation (Newcombe 2018). There has also been development towards quite general theories of the mind and body through approaches like layered neural networks (Galus 2024). Such networks already see some level of redundant connectivity for different contexts and modalities; they are not generally constrained to be sure they do not produce formal similarities in different local areas of the network. They could possibly develop towards the specific issue raised here as more work is done with similar approaches.

The reader who is interested in further application of conventional typology theory should see recent work in spatial training (Gilligan et al. 2018, 2020). This work first found that one specific type of spatial cognition was particularly important at 6–10 years old. This was then used as a target for a 5-min intervention in a follow-up study. This intervention led to effects as large as d > 0.3 on important mathematics education outcomes. This is beginning to realize the promise of spatial training, the idea that it can have a two-for-one benefit by improving spatial cognition itself as well as mathematics and science outcomes. It is no accident that this happened when applied research was directly informed by in-depth basic theory.

The obvious, yet useful, future direction is further work on an overall theory of the typology of spatial cognition. The conventional theory has both an intrinsic versus extrinsic distinction, a static versus dynamic distinction, and a third realm of symbol/metaphor that act as a separate type (Newcombe 2018). The pursuit of such a typology is still young in terms of scientific research and there may be major innovations in the near future.

In conclusion, results here help confirm that mental rotation and perspective taking reflect different cognitive processes via verification of a performance profile difference. Performance as a function of rotation magnitude has a different shape for the two tasks (linear for mental rotation, notched for perspective taking). This pattern difference was already seen in previous research (Wraga et al. 2005) and the present study confirmed this replicates even with more trials, more participants, and updated statistical controls—including a full formal verification that the profiles are different shapes rather than just having different effect sizes. This helps confirm that mental rotation versus perspective taking tasks reflect fundamentally different cognitive processes, justifying their central place in our typological theories of spatial cognition.
